# Ancient mitogenomes of Phoenicians from Sardinia and Lebanon: A story of settlement, integration, and female mobility

**DOI:** 10.1371/journal.pone.0190169

**Published:** 2018-01-10

**Authors:** E. Matisoo-Smith, A. L. Gosling, D. Platt, O. Kardailsky, S. Prost, S. Cameron-Christie, C. J. Collins, J. Boocock, Y. Kurumilian, M. Guirguis, R. Pla Orquín, W. Khalil, H. Genz, G. Abou Diwan, J. Nassar, P. Zalloua

**Affiliations:** 1 Department of Anatomy, University of Otago, Dunedin, New Zealand; 2 Department of Computational Genomics, IBM T. J. Watson Research Center, Yorktown Heights, New York, United States of America; 3 Department of Biology, Stanford University, Stanford, California, United States of America; 4 Department of Integrative Biology, University of California Berkeley, Berkeley, California, United States of America; 5 Department of Human Genetics, David Geffen School of Medicine, University of California Los Angeles, Los Angeles, California, United States of America; 6 School of Medicine, Lebanese American University, Byblos, Lebanon; 7 Department of Storia, Scienze dell’Uomo e della Formazione, University of Sassari, Sassari, Italy; 8 Department of Arts and Archeology, Lebanese University, Beirut, Lebanon; 9 Department of History and Archeology, American University of Beirut, Beirut, Lebanon; University of Florence, ITALY

## Abstract

The Phoenicians emerged in the Northern Levant around 1800 BCE and by the 9^th^ century BCE had spread their culture across the Mediterranean Basin, establishing trading posts, and settlements in various European Mediterranean and North African locations. Despite their widespread influence, what is known of the Phoenicians comes from what was written about them by the Greeks and Egyptians. In this study, we investigate the extent of Phoenician integration with the Sardinian communities they settled. We present 14 new ancient mitogenome sequences from pre-Phoenician (~1800 BCE) and Phoenician (~700–400 BCE) samples from Lebanon (n = 4) and Sardinia (n = 10) and compare these with 87 new complete mitogenomes from modern Lebanese and 21 recently published pre-Phoenician ancient mitogenomes from Sardinia to investigate the population dynamics of the Phoenician (Punic) site of Monte Sirai, in southern Sardinia. Our results indicate evidence of continuity of some lineages from pre-Phoenician populations suggesting integration of indigenous Sardinians in the Monte Sirai Phoenician community. We also find evidence of the arrival of new, unique mitochondrial lineages, indicating the movement of women from sites in the Near East or North Africa to Sardinia, but also possibly from non-Mediterranean populations and the likely movement of women from Europe to Phoenician sites in Lebanon. Combined, this evidence suggests female mobility and genetic diversity in Phoenician communities, reflecting the inclusive and multicultural nature of Phoenician society.

## Introduction

**Fig 1:**
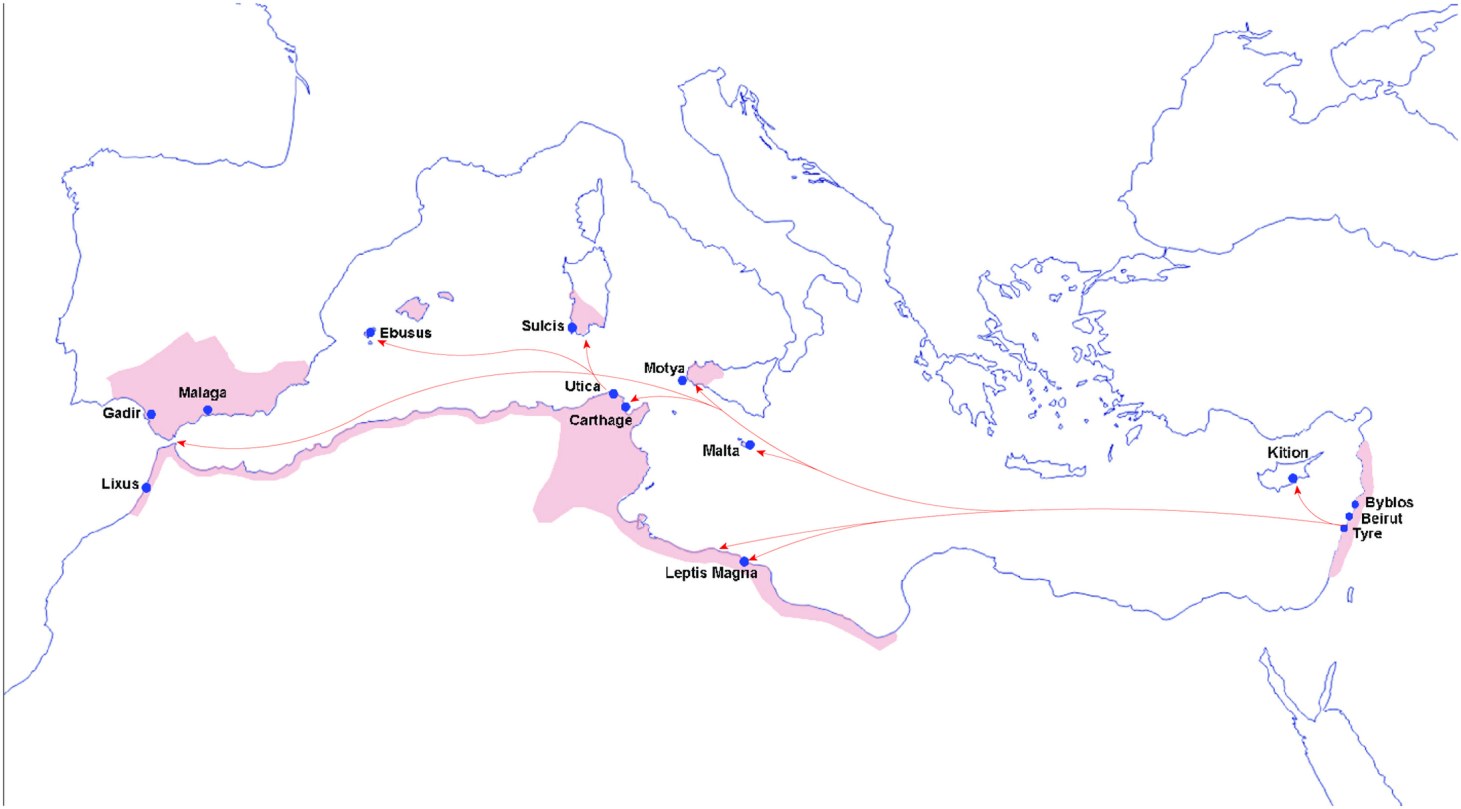
Map showing phoenician maritime expansions across the Mediterranean starting from around 800 BCE. Arrows indicate maritime movement. Blue dots indicate coastal sites and pink shaded areas indicate the extent of Phoenician settlements.

The Phoenicians, one of the great ancient civilizations, brought together East and West; they linked Asia, Europe and Africa through their trade networks and settlements, and they created the ancestral alphabet to most of the Western world today. They were skilled navigators whose trade networks extended throughout the entire Mediterranean basin, and they had taken an Egyptian-sponsored circumnavigation of Africa long before the Vikings ventured out of the sight of land [1, 2]. From their homeland in what is today Lebanon, the Phoenicians sailed extensively across the Mediterranean for trade and established settlements in Cyprus, Malta, Sicily, Sardinia, Ibiza, the Iberian Peninsula and along the North African coast, most notably, Carthage (Fig 1). Their naval dominance was respected throughout the Mediterranean, and they provided maritime support to the Persians and the Egyptians.

Despite their enduring influence, scant historical documentation attributed directly to the Phoenicians exists. Most of the Phoenician historic documents were written on papyrus, and have not survived or been discovered yet. What we do know of them is what others, the Greeks, Romans, Egyptians wrote about them. They were never a unified political state or distinct ethnicity within their homeland [3]. The emerging coastal city-states of Byblos and Sidon thrived as maritime trade centers during the 3^rd^ and 2^nd^ millennia BCE and later, the city of Tyre. During the 1^st^ millennium BCE their occupants were referred to by the Greeks, and thus today, as the Phoenicians (from the Greek, *Phoiníkē*, or purple country) in reference to their production of the valuable purple dye used in textile production. Prior to this, however, the region was occupied by Canaanites who, due to political events in the north, south and east, were confined to the thin coastal strip between the steep, cedar covered mountains of Lebanon and the eastern Mediterranean [3]. This coastal isolation meant that Phoenicians could only expand westwards, and this they did. Targeting sources of valuable metals including silver and tin, they established settlements across the Mediterranean and dominated maritime trade networks for centuries.

Starting in the middle of 8^th^ century BCE the Assyrians waged war on the Phoenicians and soon after they brought them under their yoke. With the decline of Phoenician influence in the east, Carthage, a city that was settled by Phoenicians fleeing Tyre in 813 BCE, became the centre of western Phoenician, or Punic, dominance until its destruction in 146 BCE by the Romans in the 3rd Punic War. Carthage, like many of the early Phoenician cities around the Mediterranean, adopted many of the rituals and features of their homeland.

The island of Sardinia, like Cyprus, Malta, Sicily, and Ibiza, was a significant location in the early Phoenician trade routes to the Iberian and North African coasts, and later in the Punic interaction sphere. Phoenician settlements were established primarily along the south and west coast of Sardinia, with two of the earliest located at Sulcis and, slightly inland, Monte Sirai. As was common in Phoenician Sardinia, Monte Sirai was built next to previous Nuragic tower used by the first inhabitants like a shrine. The necropolis at Monte Sirai was one of the largest Punic burial sites in Sardinia and was used between the end of 7^th^ and the first half of 4^th^ century BCE. This period of use coincides with the Carthaginian dominion in Sardinia (starting at the end of 6^th^ century BCE).

Genetic research on modern populations in Lebanon and the Levant, the homeland of the Phoenicians, shows that much of the genetic structure of the region today is influenced by historic migrations associated with the spread of major religions–Christians, Muslims and Druze [4]. Research on the Y chromosome DNA of men currently living in locations of historic Phoenician influence from across the Mediterranean, compared with men from nearby locations with no evidence of Phoenician contact, has identified likely Phoenician Y chromosome markers. These markers were present in more than 6% of the men living in Phoenician-influenced locations around the Mediterranean and in more than 30% of Lebanese men [5]. Comparative analyses of uniparental genetic markers in modern Lebanese indicate different genetic histories between males and females, with significantly more European lineages evident in the maternally inherited mitochondrial DNA (mtDNA) than observed for the paternally inherited Y chromosome [6]. When these European-derived lineages were integrated into the Lebanese population is not yet fully resolved. If identified, mitochondrial markers associated with Phoenician settlement around the Mediterranean may help document the movement of women within the Phoenician networks. They can also be used to indicate how much integration with indigenous inhabitants occurred in Phoenician settlements. We now have the opportunity to investigate the genetic and cultural history of the Phoenicians and their spread across the Mediterranean through the analysis of ancient DNA (aDNA) from skeletal remains recovered from pre-Phoenician, Phoenician and Punic archaeological sites across the Mediterranean.

Here we describe the results of analyses of complete mitochondrial genomes of ancient samples recovered from three Phoenician sites in Lebanon and Sardinia and one slightly earlier Middle Bronze Age site in Lebanon. We compare these results to modern mitochondrial genome data from Lebanese populations and recently published, pre-Phoenician ancient Sardinian mitogenome data to identify possible Phoenician/Punic-introduced mitochondrial haplotypes and attempt to reconstruct the history of interactions and the Phoenician genetic influence on modern population diversity.

## Materials and methods

### Archaeological sites and samples

A total of 28 ancient tooth samples were obtained for aDNA analyses from four archaeological sites in Lebanon, BEY 197, BEY 198, Saifi 477, and Tell Fadous-Kfarabida, and one site, Monte Sirai, in Sardinia. Permits and approval for this study were obtained from the office of the Director General of Antiquities in Lebanon (permit number 4290, 6 November 2015) for all Lebanese samples and from the Ministry of Culture and Tourism (prot. No. 5844, 17 March 2016), Superintendent of Archaeology in Cagliari) for the Monte Sirai samples. The Sardinian samples are held in the laboratory of archaeology "Sabatino Moscati" located at the Segni's Palace, University of Sassari, and the Lebanese samples are stored with the Directorate General of Antiquities, in Beirut, Lebanon.

Two closely related sites, BEY 197 and BEY 198, were excavated in 2012 and 2013, and are located at the center of the Lebanese capital, Beirut. The archaeological context of the samples we studied from BEY 198 relates to the late Phoenician, Achaemenid period (539–330 BCE). In addition to the stratigraphy, a pilgrim flask, a dish, a pitcher, a gold ring and red carnelian beads were found in the tomb, enabling accurate dating of this archaeological context. This tomb, heavily demolished during construction works in the 2nd century BCE, contains skeletal remains belonging to several individuals, most probably four persons, including one adolescent and a child. The remains were found in a funerary chamber dug in the rock [7]. The excavated grave in BEY 197 is an inhumation burial found in a high state of fragmentation with extensive penetrating erosion. The single skeleton was laid inside a rectangular cut (pit) in bedrock with an (E-W) orientation and was covered by a yellowish-reddish, compact to weakly cemented, cobble/sand fill. The provisional dating assigned to this grave ranges between the 4th and the 1st century BCE [8].

The Saifi 477 site is also located in Beirut and was excavated between 2013 and 2014 [9]. The skeletal remains in this site were found in pits excavated in the geological substrate. Pits containing children and not adults were lined and covered with pebbles. The deceased were placed on their backs and their limbs were in an extended position. Two of the skeletons from which our samples were obtained were accompanied with funeral material: an adult wore a thick copper alloy bracelet around the right leg and a child with a copper alloy bracelet around each ankle. All these burial rituals are consistent with a 5^th^—4^th^ century BCE date.

The fourth site in Lebanon was at Tell Fadous-Kfarabida, a town situated 2 km south of the coastal town of Batroun, and revealed the presence of three Middle Bronze Age tombs. Excavations on this site started in 2007 and the skeletal remains for this study were unearthed between 2007 to 2010. The pottery from the three tombs is quite homogeneous and can be dated to the end of the Middle Bronze Age I (~1800 BCE). This dating is also supported by a scarab found with one of the burials which dates to the late 12th to 13th Egyptian Middle Kingdom Dynasty [10, 11].

The archaeological site of Monte Sirai is a hill-top settlement located in Carbonia, south-western Sardinia, Italy. The site is comprised of three large sectors: the main sector of the settlement, the “acropolis”, located in the southern portion of the hill; the sacred place, or “tophet”, located in the northern portion of the site, and the large necropolis located in the valley separating the settlement from the tophet (S1 Fig). Archaeological evidence indicates that Monte Sirai was founded by Phoenicians in the last quarter of 8^th^ century BCE, and totally abandoned during the 1^st^ century BCE. The necropolis contained several tomb types including inhumations of single and multiple individuals, primary cremations, *enchytrismoi* (child burials in transport amphorae) and semi-combustions. The first excavations of the site were carried out in the 1960s, and again in the 1980s [12], though systematic excavations, involving a large graveyard area, began in 2005 and are on-going [13, 14]. Samples were obtained during the 2015 field season from a range of burial contexts including three single pit burials; one jar burial; and two group burials, one containing four, and the other, two individuals, all dating from the period between the end of 6th and the first decades of 4th century BCE.

### Sample descriptions

A total of 16 ancient tooth samples were obtained from the four archaeological sites in Lebanon: 2 from BEY 197, 8 from BEY 198, 4 from Saifi and 2 from Tell Fadous-Kfarabida. From Sardinia, 12 tooth samples were obtained from burials recovered during the 2015 excavations at the Punic necropolis of Monte Sirai.

The modern Lebanese DNA collection consisted of 87 samples that were collected by our team from volunteers representing the various communities across Lebanon, who provided information about their place of birth and the geographical origins of three generations of Lebanese maternal ancestry. A written informed consent was signed and obtained by each participant prior to recruitment. The study protocol and the informed consent form were approved by the IRB of the Lebanese American University. Study methods were carried out in accordance with the principles of the Declaration of Helsinki.

Complete mitogenome data from 21 ancient, pre-Phoenician samples from Sardinia used for comparative analyses here are from Olivieri et al. [15].

### DNA extraction, hybridization capture and sequencing

All archaeological samples were sent to the purpose-built ancient DNA facility [16] at the University of Otago for processing. DNA extraction, library preparation and in-solution hybridization capture of the mitochondrial genomes were conducted using methods described previously [17]. Post-capture libraries were purified, quantified, pooled and were then run on an Illumina MiSeq sequencing platform in a 2 x 75 base paired-end run.

### Data Analyses

#### Modern DNA sequence processing

The complete mitochondrial genomes for 87 modern Lebanese samples were prepared and sequenced using methods described previously [17].

#### Ancient DNA sequence processing

Ancient DNA sequences were also processed following the protocols described previously [17]. Specifically, ancient sequences were subjected to AdapterRemoval [18], where sequencing adaptors, reads with stretches of Ns, bases with quality scores of <30 and short reads (<25 bp) were removed. Additionally, where paired end fragments overlapped by at least 11 base pairs, reads were merged. Merged and unmerged reads were aligned separately with Burrows-Wheeler Aligner (BWA) using recommended ancient DNA settings in which seeding was disabled (-l 1024), gap opens was set to 2 (-o 2) and maximum edit distance was set to 0.03 (-n0.03) [19]. To test for contamination with laboratory reagents, all reads were mapped to a composite reference genome constructed using the revised Cambridge Reference Sequence (rCRS) for humans [20], along with the reference mitochondrial genomes for cow (*Bos taurus*), pig (*Sus scrofa*) and chicken (*Gallus gallus*) (GenBank refs NC_006853, NC_0012095.1 and NC_001323.1). The contamination ratio was determined by calculating the ratio of all reads with mapping quality > = 20 for each reference. We determined the contamination ratio by calculating the ratio of all reads with a mapping quality > = 20 for each reference, compared to those for the human CRS.

PCR duplicates were marked and removed from unmerged reads using Picard tools 1.92 (http://broadinstitute.github.io/picard/), and from merged reads using a python script developed by Fu et al. [21]. MapDamage (v2.0.2–9) [22] was used to identify characteristic aDNA damage patterns, with the ‘-rescale’ option to lower the quality score of likely damaged sites. Thiamines (T) found at the 5’end of a read and guanines (G) found at the 3’ ends within the first two bases were rescaled to 0 for their quality scores. Modern human contamination levels were assessed using the ContamMix package with a set of 311 representative modern human mtDNA sequences [23].

Variant call files (VCFs) were generated for all reads that mapped to the human reference genome using the GATK Haplotype Caller (v3.5) [24] with settings specific for haploid genomes and were filtered for mapping quality (<20) and bases that were covered by fewer than 3 reads. We only analysed sequences having a minimum of 96% coverage. VCF files were converted to whole-sequence FASTA files using in-house commands and GATK’s FastaAlternateReferenceMaker. Coverage plots were created for all reads using R software [25], and plots of fragment length were created from merged reads with Picard’s CollectInsertSizeMetrics (Picard tools 1.92, http://broadinstitute.github.io/picard/). Consensus sequences were created (including indels) and these were deposited in GenBank. Sequences were assigned to haplogroup using Haplogrep [26] with Phylotree build 17. All reads generated have been submitted to the NCBI Sequence Read Archive (SRP123440), identified by lab sample number.

#### Phylogenetic analyses

Median-Joining network analysis was applied to all ancient sequences using POPART [27] with default settings. Median-Joining networks were constructed by combining minimum-spanning trees within a single network.

Discriminant analysis of principal components (DAPC) was used to investigate clustering of related individuals. DAPC is a multivariate analysis used to extract information of genetic relationships for between- and within-species/population sequence data [28]. The DAPC approach is integrated into the R package *adegenet* [29]. We used the *fasta2DNAbin* function provided by *adegenet* to load the sequence data into R. We then transformed the data to a genind file using DNAbin2genind (DAPC input format). The principal components (PC) and discriminant functions to retain were chosen using the plots created by the DAPC function (*dapc*). All R packages used for the cluster analysis can be freely downloaded at: http://cran.rproject.org/web/packages/.

To investigate the phylogenetic relationships between the ancient mitogenomes that we obtained and modern population data, Maximum likelihood (ML) trees were constructed using PhyML [30]. Modern sequences for each haplogroup identified in our ancient samples were downloaded from Genbank. The most appropriate nucleotide substitution model was determined using jModeltest [31].

## Results

The haplogroup assignments and Genbank accession numbers for the 87 new modern Lebanese mitogenomes are shown in S1 Table. Coverage data and variable sites for all modern Lebanese samples are shown in S2 Table, and coverage data, ContamMix data and variable sites for all ancient samples processed are shown in S3 Table.

### Sequence authenticity of aDNA

The results of the tests for contamination from laboratory reagents indicated that very few reads mapped to any of the possible reagent contaminators of cow, pig, dog or chicken. The results of the assessment of the contamination from modern human DNA using the program ContamMix also indicate minimal modern contamination and are shown in S3 Table.

From the 16 ancient Lebanese samples processed, a total of four complete ancient mitogenomes from three archaeological sites were considered acceptable in terms of their coverage (at least 3x), and showing appropriate damage patterns and no significant level of contamination, resulting in a success rate of 25%. From Monte Sirai, Sardinia, 10 complete mitogenomes were generated from the 12 samples processed, resulting in a success rate of 83%. This difference in success rates between the two regions is likely related to several factors impacting DNA preservation including temperature, humidity and post-excavation treatment [32]. Of the four Lebanese samples for which we were able to obtain acceptable full mitogenomes, one was from Tel Fadous-Kfarabida, one from BEY 197 and two were from BEY 198. All three of these sites were excavated in the last decade. The Lebanese samples were not washed in the field or lab, but they were not collected or stored specifically with the intention of future aDNA analyses. The soils from the Saifi site appeared to be moist, which may explain the poor results (0/4) from that site. Previous attempts to recover aDNA results from samples from a wet part of site BEY 198 (next to a well) were all unsuccessful (unpublished data). The Monte Sirai samples were all recently (2015) excavated and collected with the expressed purpose of aDNA analysis. Most of the graves were pits dug into the tuff (natural rock soil) that were closed, covered by stone slabs, and one sample, MS10584, was from a jar burial (*enchytrismoi*). These features may have provided protection from the elements resulting in better DNA preservation.

The median average read depth across the mitochondrial genome for the 14 ancient samples was 22.7 (S2 Fig). Sardinian samples had a much wider range of coverage (range of mean coverage was 4.8 to 1321.2, while the range for Lebanese samples was 4.8 to 49.8). However, the amplicon length between the two groups was not significantly different (Welch two-sample t-test, t = 0.05, df = 5.42, p = 0.96) (S3 Fig). Damage patterns were consistent with those expected from aDNA [33] for all samples included in analyses (S4 Fig).

S5 Fig shows our 14 ancient samples placed within the maximum parsimony tree presenting a phylogeny of 438 published ancient mitogenomes published in S5 Fig of Olivieri et al. [15].

We aimed to identify regionally informative mtDNA haplogroups to use as indicators of historical Phoenician ancestry. Populations that diverged from one-another carry uniparental lineages that accumulate mutations randomly, yielding increasing genetic distances between those populations. The induced geographic correlation with genetic distance will be visible using median-joining network techniques [34], and in the distance information contained in the ancestrally-informative components [35, 36] in the singular value decomposition [37] of SNP data. Interpretation is subject to the caveat that principal component analysis identifies any leading alignments marking the largest distances among samples; connection to migrations requires care [38]. Statistics measuring the discriminating power of principal components between populations, such as DAPC [28], serve to test the isolation between populations. The strongest signals differentiating these populations will tend to be the oldest, since these yield the largest genetic differences.

Table 1 shows the haplogroup assignments, site information, dates and Genbank accession numbers for all ancient samples used in the analyses. Variable sites identified for modern and ancient samples sequenced are shown in S2 and S3 Tables. All haplogroup assignments were assigned according to phyloTREE build 17 [39]. The coverage plots, read lengths and damage patterns for the 14 ancient samples processed for this study are shown in S2, S3 and S4 Figs.

**Table 1:** Haplogroup assignments, dates, locations and Genbank accession details of all aDNA samples included in analyses.

Sequence ID	Haplogroup	Age/Date	Country	Site/cotext	GenBank ID	Reference
**MS10560**	**T2b3+151**	**4th-1st c BCE**	**Lebanon**	**Bey197 Context 302**	**KY797250**	**This study**
**MS10562**	**H**	**539–330 BCE**	**Lebanon**	**Bey198 Cxt 529**	**KY797251**	**This study**
**MS10565**	**R0a2n**	**539–330 BCE**	**Lebanon**	**Bey198 Cxt 5048**	**KY797253**	**This study**
**MS10575**	**H34**	**~1800 BCE**	**Lebanon**	**FAD10 290/305 434**	**KY797254**	**This study**
**MS10577**	**H5d**	**early 5th c BCE**	**Sardinia**	**Monte Sirai Tomb 351**	**KY797255**	**This study**
**MS10578**	**N1b1a5**	**end of 5th c BCE**	**Sardinia**	**Monte Sirai Tomb 344**	**KY797256**	**This study**
**MS10579**	**J1c+16261+189**	**end of 5th c BCE**	**Sardinia**	**Monte Sirai Tomb 352**	**KY797257**	**This study**
**MS10580**	**J1c+16261+189**	**end of 5th c BCE**	**Sardinia**	**Monte Sirai Tomb 358**	**KY797258**	**This study**
**MS10581**	**W5**	**end of 5th c BCE**	**Sardinia**	**Monte Sirai Tomb 355**	**KY797259**	**This study**
**MS10582**	**H3**	**end of 6th c BCE**	**Sardinia**	**Monte Sirai Tomb 346**	**KY797260**	**This study**
**MS10584**	**H+16311**	**5th c BCE**	**Sardinia**	**Monte Sirai Tomb 349**	**KY797261**	**This study**
**MS10585**	**H1e1a6**	**end of 5th c BCE**	**Sardinia**	**Monte Sirai Burial 347**	**KY797262**	**This study**
**MS10587**	**X2b+226**	**end of 5th c BCE**	**Sardinia**	**Monte Sirai Tomb 354**	**KY797263**	**This study**
**MS10588**	**H1bn**	**end of 5th c BCE**	**Sardinia**	**Monte Sirai Tomb 357**	**KY797265**	**This study**
**MA100**	**T2c1d**	**4800–4450 calBP**	**Sardinia**	**Scab’e Arriu M**	**KY399143**	**Olivieri et al 2017**
**MA104**	**K1a**	**4520–4410 calBP**	**Sardinia**	**Bingia’ e Monti**	**KY399144**	**Olivieri et al 2017**
**MA108**	**HV0j**	**4420–4260 calBP**	**Sardinia**	**Pardu Jossu**	**KY399145**	**Olivieri et al 2017**
**MA110**	**T2b3**	**3170–3000 calBP**	**Sardinia**	**Ingurtosu Mannu**	**KY399146**	**Olivieri et al 2017**
**MA112**	**V**	**3210–3010 calBP**	**Sardinia**	**Is Arutas**	**KY399147**	**Olivieri et al 2017**
**MA115**	**H3**	**3330–3080 calBP**	**Sardinia**	**Mont ‘e Prama**	**KY399148**	**Olivieri et al 2017**
**MA138**	**H5a**	**3180–3000 calBP**	**Sardinia**	**Is Arutas**	**KY399149**	**Olivieri et al 2017**
**MA73**	**J2b1a**	**6180–5950 calBP**	**Sardinia**	**S'isteridolzu**	**KY399150**	**Olivieri et al 2017**
**MA74**	**J1c3**	**6190–6000 calBP**	**Sardinia**	**S'isteridolzu**	**KY399151**	**Olivieri et al 2017**
**MA76**	**H1**	**6190–5960 calBP**	**Sardinia**	**Noeddale**	**KY399152**	**Olivieri et al 2017**
**MA77**	**H1e1**	**5650–5490 calBP**	**Sardinia**	**Noeddale**	**KY399153**	**Olivieri et al 2017**
**MA78**	**H3**	**4090–3900 calBP**	**Sardinia**	**Su Asedazzu**	**KY399154**	**Olivieri et al 2017**
**MA79**	**U5b2b3**	**6180–5930 calBP**	**Sardinia**	**Longu Fresu**	**KY399155**	**Olivieri et al 2017**
**MA81**	**K1b1b1**	**3970–3720 calBP**	**Sardinia**	**Su Cannisoni**	**KY399156**	**Olivieri et al 2017**
**MA82**	**H1**	**3550–3380 calBP**	**Sardinia**	**Su Cannisoni**	**KY399157**	**Olivieri et al 2017**
**MA85**	**H1e1a**	**3970–3830 calBP**	**Sardinia**	**Stampu Erdi**	**KY399158**	**Olivieri et al 2017**
**MA86**	**U5b2a**	**4090–3890 calBP**	**Sardinia**	**Monte Gastea**	**KY399159**	**Olivieri et al 2017**
**MA87**	**J2b1a**	**3140–2870 calBP**	**Sardinia**	**Asedazzu**	**KY399160**	**Olivieri et al 2017**
**MA88**	**U5b2b5**	**4300–4010 calBP**	**Sardinia**	**Su Asedazzu**	**KY399161**	**Olivieri et al 2017**
**MA89**	**K1b1a1**	**5320–5060 calBP**	**Sardinia**	**Cannas di Sotto Tomb 12**	**KY399162**	**Olivieri et al 2017**
**MA92**	**J2a1a1**	**4790–4440 calBP**	**Sardinia**	**Seddas de Daga**	**KY399163**	**Olivieri et al 2017**

### Phylogenetic analyses

**Fig 2:**
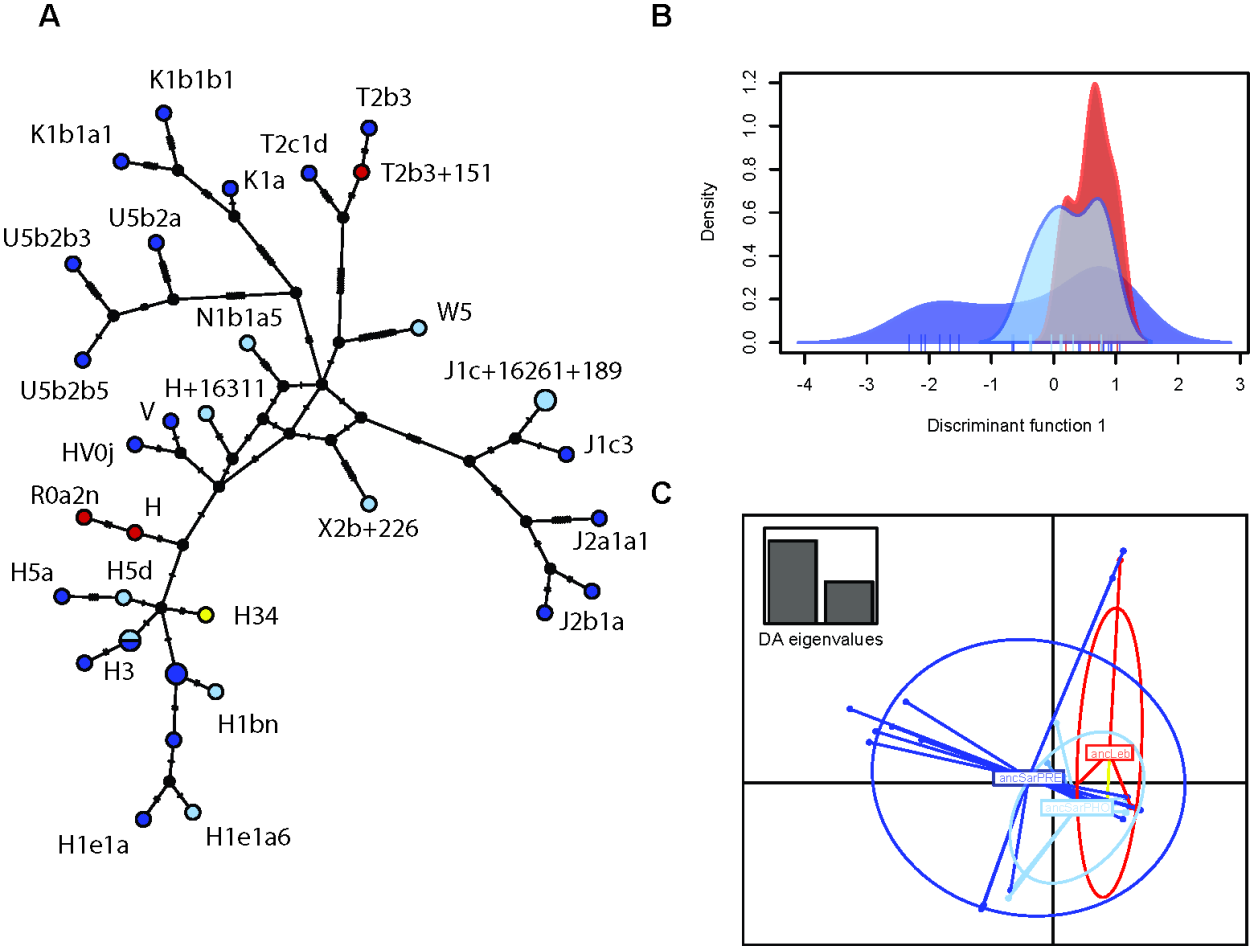
Median-Joining network and DAPC analysis. A) Median-Joining network of all ancient mitogenomes. Dark blue circles are pre-Phoenician samples from Sardinia [15], light blue circles are Phoenician samples from Sardinia and red circles are Phoenician samples from Lebanon and the orange circle represents the pre-Phoenician sample from Lebanon. The number of mutations separating sequences are shown as dashes on the branches B) DAPC analysis retaining 4 PCs and 1 DA eigenvalues; and C) DAPC analysis retaining 4 PCs and 2 DA eigenvalues. Colours are the same as in 1A.

The Median-Joining network incorporating all of our ancient mitogenomes from Sardinia and Lebanon with the pre-Phoenician Sardinian data from Olivieri [15], and the results of the DAPC analyses of these same samples are shown in Fig 2. We only found one haplotype (H3) shared between pre-Phoenician and Phoenician era samples from Sardinia and this can be seen in the network analysis (Fig 2A). However, we do not find many mutations separating haplotypes from all the three groups, particularly those in haplogroup H. Two clades (the K1 and U5 haplogroups) contain samples from pre-Phoenician Sardinians only, whereas the W5, N1b1a5 and X2b clades contain only Phoenician Sardinians, and appear to be distant from any pre-Phoenician Sardinian samples and thus we suggest that these are likely Phoenician samples. The T2b3 sample from Beirut is only two mutations removed from a pre-Phoenician Sardinian sample and does not appear to be an indigenous Lebanese lineage but rather a foreign introduction to the Beirut Phoenician population. We carried out DAPC analyses to investigate genetic structuring within our sampling. DAPC performs discriminant analyses (DA) on principle components (PC). Generally, DA resolves between population relationships while ignoring within population variation [28]. DAPC uses PCA to resolve within population variation and then performs a DA to resolve between population structure [28]. The two DAPC plots (one discriminant function and two discriminant functions) support the pattern found in the network analysis, identifying overlapping signatures of Lebanese samples with the Sardinian Phoenicians, with a clear pre-Phoenician component of the population (Fig 2B and 2C). We see that most Phoenician-era samples cluster closely together with pre-Phoenician samples.

**Fig 3:**
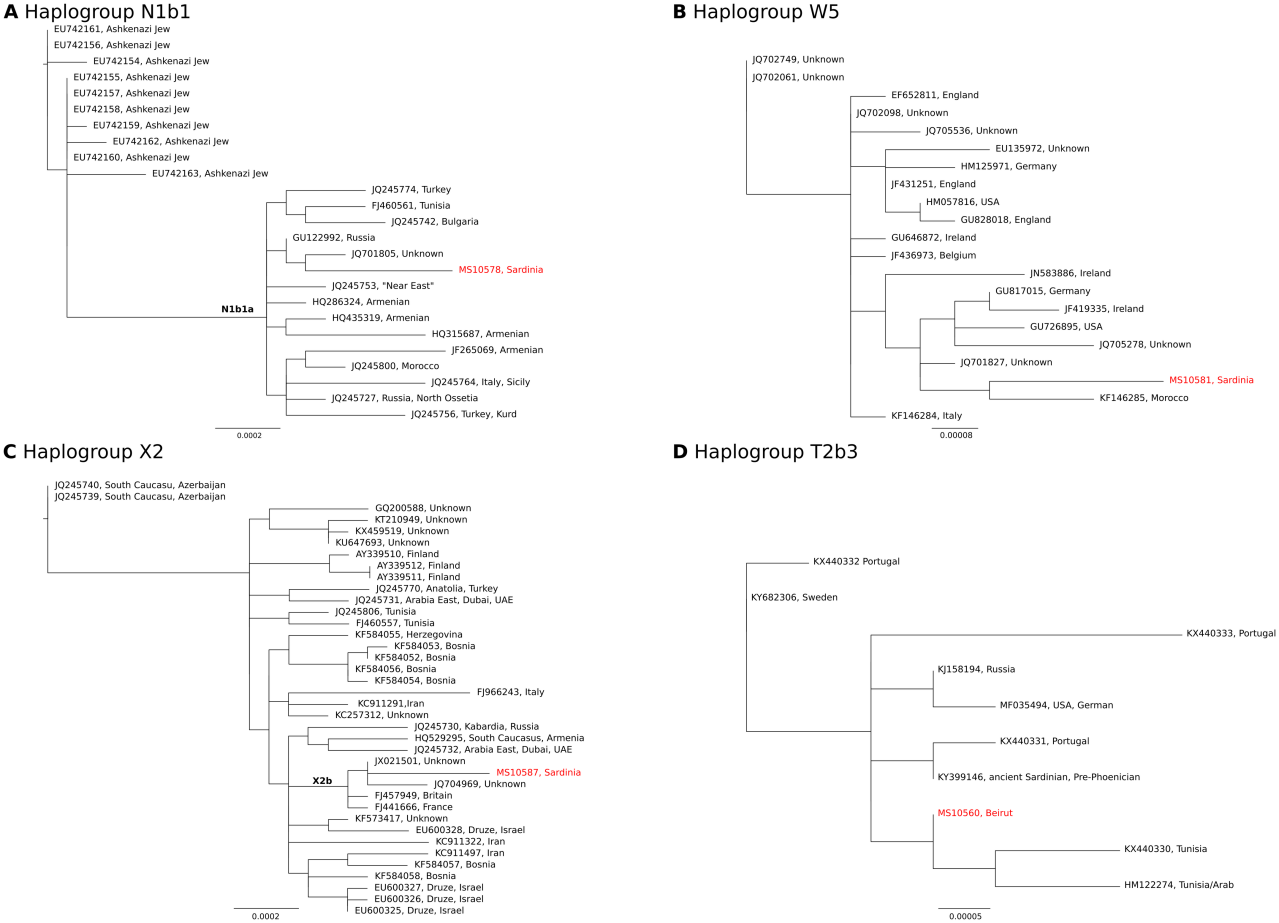
Maximum likelihood trees for what appear to be introduced ancient Phoenician haplotypes and publicly available sequences for the corresponding mitochondrial haplogroups. All samples are modern except for sample KY399146 (pre-Phoenician Sardinian; from Olivieri et al. 2017) and ancient samples from this study (MS10578, MS10581, MS10587 and MS10560). Each node is annotated with the GenBank accession number and the sample location, where known. Sequences from this study are highlighted in red text. A. N1b1 (including MS10578; N1b1a branch is labeled with bold text), B. W5 (including MS10581), C. X2 (including MS10587; X2b branch is labeled with bold text) and D. T2b3 (including MS10560).

Four of the haplotypes identified in our ancient Phoenician samples stand out as being possible candidates for Phoenician introductions as they appear to be foreign when compared to both modern and ancient data from the location in which they were found. The ML trees for haplogroups N1b1, W5, X2, which were found in our Phoenician samples from Monte Sirai, and T2b3 which appears to be a foreign introduction to the BEY 197 site in Lebanon, are shown in Fig 3. The most appropriate nucleotide substitution model was determined to be HKY for haplogroups N1b1, W5 and T2b3 and TrN for the X2 haplogroup.

### Archaeological context and relatedness of group burials in Monte Sirai

The archaeological interpretation of the group burials in Monte Sirai is that they could represent family burials. We found that one group burial, containing four individuals, included two 6 to 12-year-olds who had near-identical J1c haplotypes (see S3 Table). MS10579 and MS10580 were identified as J1c with SNPs at positions 16261 and 189. They also shared two novel mutations, 310.1C and 7154G. The remaining differences between these two haplotypes are four C to T transitions in sample MS10579, which are very likely to be the result of damage, and two missing SNPs which are due to reduced coverage in this region. It is therefore possible that these two were indeed siblings or otherwise maternally related, for example, cousins. The other two burials in that group had the two non-local lineages: another 6 to 12-year-old with the N1b1a5 and a young adult female, 16 to 22 years of age, with the W5 haplotype. While this indicates that there is not a maternal connection between these four individuals who were buried together, it is possible that they are family members related through paternal connections. The other group burial contained two individuals, an 8 to 14-year-old with an X2b haplotype and a young adult male with an H1bn mitogenome. Analyses of more burials from Monte Sirai and possible extended analyses of Y chromosome or autosomal data are necessary to further test the relatedness of individuals buried in Monte Sirai.

## Discussion

The aim of the study was to seek mitochondrial DNA markers recovered from ancient samples that can be associated with Phoenician origins and ancestry, enabling us to track Phoenician mobility and genetic impacts of settlement, in this case, in Sardinia. We collected ancient samples from documented Middle Bronze Age and Phoenician burials in Lebanon, and from Monte Sirai, Sardinia, one of the largest Phoenician-Punic necropoli in the central Mediterranean.

Without ancient DNA, a Phoenician introduced signature can be difficult to distinguish from earlier Near Eastern lineages that spread during the Neolithic expansion to European sites that were later colonized by the Phoenicians, like Monte Sirai. It is expected however, that Neolithic expansions would spread lineages broadly across the landscape, where Phoenician signatures are likely to be restricted to locations of well-established Phoenician contact [5]. Due to the relative isolation of Sardinia and the unusual genetic signature of its population, modern Sardinians are often presented as the best representation of the early Neolithic population that expanded from the Near East into Southern Europe [40, 41]. The recent study of Olivieri et al. [15], which provides mitogenomes for 21 ancient samples and over 2000 new modern Sardinian samples, identifies numerous Sardinian-specific haplogroups including a few lineages they argue are likely indicative of pre-Neolithic settlement of the island. Their ancient samples date to between 6000 and 3000 years calBP and thus provide a valuable snapshot of the pre-Phoenician population of Sardinia.

While the later Phoenician influence in Sardinia was likely via Carthage, as indicated by the archaeological evidence from Monte Sirai [13, 14], we still might expect to see the appearance of new Near Eastern and/or North African mitochondrial lineages in the burials from the site given that Carthage was settled directly from Tyre. The appearance of Phoenician introduced markers in Sardinia might also be inferred by analyses of the estimated TMRCAs of shared haplogroups in modern populations in Lebanon and Sardinia that potentially differentiate them from older Neolithic signatures spread throughout Europe.

### Haplogroup H

The most common haplogroup seen in our ancient Lebanese and Sardinian samples was the superhaplogroup H, identified in 7 of 14 samples (50%). Two of the four ancient Lebanese samples belong to haplogroup H (sub-groups H and H34) and a third belongs to the sister clade, R0. Five of the Monte Sirai samples (50%) were identified as having H haplotypes (H+16311, H1e1a6, H1bn, H3, and H5d). Haplogroups H1, H3 and H5 are all thought to have a Southwest European origin and to have spread from there after the LGM [42]. There were no shared haplotypes between the ancient samples of Lebanon and those from Monte Sirai. However, all sequences belonging to haplogroups H and R0 (ancient samples of Lebanon and Sardinia, and Monte Sirai) are very closely related as they are separated by only few mutations (Fig 2A). Olivieri et al. [15] also report high levels of H subgroups in their ancient samples (38%) with several closely related lineages to our Monte Sirai samples (HV0j1, H1, H1e1, H1e1a, H3, H3u and H5a).

In modern populations, the frequencies of haplogroup H range between 40–50% in Europe and about 20% in Southwest Asia. Subclades H1 and H3, the major subclades of this haplogroup, are almost exclusively European with limited penetration of H1 in the Near East, suggesting a European origin with estimated ages of 14Ky-16Ky and 9Ky-11Ky respectively [43]. Haplogroups H1 and H3 are the most common found in modern Sardinians, with several Sardinian specific lineages recently identified [15]. No H3 lineages were found in our ancient or modern Lebanese samples. Its presence in Sardinia in ancient samples (both pre-Phoenician and Phoenician), combined with the diversity seen in the modern Sardinian population is consistent with at least a Neolithic introduction to the island if not pre-Neolithic [15]. Haplogroup HV is also an ancient European lineage, likely originating in the Mediterranean region during the LGM and has been identified in early Neolithic remains from Spain [44]. However, to date, most studies on haplogroup H and its subgroups reveal a very complex tree structure and more mitogenome data is needed before any conclusion can be made with certainty about the origin and date of the H subgroups [45].

### Haplogroup J1c

Two samples from Monte Sirai (MS10579 and MS10580) have J1c haplotypes with the addition of two previously identified mutations at positions 189 and 16261 and a unique mutation at 7154. This full motif was not found in any of our modern Lebanese samples, though we did find a J1c with the 16261 mutation, and one J1c11a haplotype. Several J1c sub-types are seen in modern Sardinians and a J1c3 was identified in one pre-Phoenician sample dating to around 4000 BCE [15]. Haplogroup J is thought to have arisen in West Asia sometime around 45 Ky Pala et al. [46] suggest that during the LGM, haplogroup J sub-groups arose in the Near Eastern refugia, though since J1c is rare in the Near East today, it may have first emerged in southeast Europe. A J2b1 haplogroup was recently identified in an early Mesolithic (8227–7596 BCE) sample from Sardinia [47]. J1c has not yet been found in any ancient Mesolithic samples in Europe, but Mathieson et al. [48] have reported J1c haplotypes in samples from Turkey, Hungary and Germany dating from 4000 BCE and in Spain from 2000 BCE. Allentoft et al. [49] found a J1c1b in a Bronze Age sample from Italy. It is clear that J1c was present in Sardinia prior to the arrival of Phoenicians, and possibly prior to Neolithic expansions.

### Haplogroup N1b1a5

The presence of one sample from Monte Sirai, MS10578, a 6 to 12-year-old child that has an N1b1a5 haplotype is of particular interest. Haplogroup N lineages are rare in modern Sardinians. Recently, however, four individuals were identified carrying haplogroup N1b1a9, which appears to be a Sardinian specific haplogroup [15]. The coalescence ages of this haplogroup are estimated to be 7.3–9.4 Ky, so this may have been a pre-Neolithic introduction to the island. N1b1a5, however, is more recent (Olivieri et al. [15] Fig 3), dating to 2.5 Ky, which aligns nicely with a Phoenician/Punic introduction. Brandt et al. [45] suggest that N is a marker of Western European Hunter Gatherers as it has been found in Mesolithic samples from Portugal and a Palaeolithic sample in Southern Italy. Ancient samples with haplogroup N1a have been found in early Neolithic sites from Spain and Germany [41], but N1b has not been recorded in Neolithic samples outside of the Levant with two exceptions from Anatolia dating to 6500–6200 BCE [48, 50] and between 7500–5800 calBCE [51]. N1b is a relatively common haplogroup in Lebanon, with 9 of the 87 (10%) modern samples we sequenced carrying N1b1a subtypes. While we have not found any N1b in our ancient Lebanese samples, it is not unlikely that this haplogroup was introduced to Sardinia via Phoenician contact, either directly from the Levant or via Phoenician/Punic settlements in North Africa, for example Carthage. Fig 3A shows that N1b1a lineages have been identified in modern Tunisians [52, 53] and in a modern Moroccan [52], as well as in a modern individual from Sicily, another island with known Phoenician settlement [54].

### Haplogroup W5

Haplogroup W is thought to have evolved during the LGM around the Caspian Sea region. Its oldest subgroup, W1 has been found in one individual from the Neolithic site of Barcın, in Anatolia dating to 6500–6200 BCE. Haplogroups W1, W3 and W6 have been found in late Neolithic and early Bronze Age sites in Germany and Russia [48] and W1 has been identified in a Neolithic sample from the Iberian Peninsula [55]. W5 has yet to be identified in Mesolithic or Neolithic remains in Europe or in any ancient samples from the Levant [48, 50]. While haplogroups W1, W3 and W10 are present in modern Sardinians [15], the archaeological sample from Monte Sirai, MS10581, a young female included in a group burial, is, as far as we know, the first W5 identified in Sardinia. The age of W5 has been estimated to be 12.2 Ky [56], and while W5 is most commonly found in Northern Central Europe and Britain today (Fig 3B), a basal W5 lineage was identified in a Moroccan Berber [56], which clusters most closely with our Phoenician sample. Our ancient result from Monte Sirai is indeed significant and establishes a minimum date of late 5^th^ century BCE for haplogroup W5 in the Mediterranean region and, given the Phoenician trade networks, could explain the presence of W5 in North Africa. It is possible that haplogroup W5 got into the Mediterranean via the Phoenician tin trade with Britain or Ireland, though only further aDNA work can confirm this hypothesis.

### Haplogroup X2b

Sample MS10587 from Monte Sirai belonged to haplogroup X2b, with an extra mutation at position 226C. Mathiesen et al. [48] report this same signature in a sample (I1499) from Garadna, Hungary dating to 5210–5010 calBCE (see S5 Fig). X2b has also been recorded in an early Neolithic sample from Revenia, Greece, dated to 6438–6264 calBCE [57]. Haplogroup X is relatively rare in Europe, generally found at frequencies of less than 1%. The highest frequencies of X in Europe are reported in Catalonia, the Pyrenees and southern Portugal, at about 2.5%. It is found at relatively high frequencies in Druze from the Levant, where it reaches frequencies of up to 15%, including subtype X2b (Fig 3C), though we did not identify X haplotypes in any of our modern Lebanese samples. It is possible that X2b was a Phoenician haplogroup introduced to Sardinia either directly from Lebanon or via North Africa, though an earlier, early Neolithic introduction, perhaps via a maritime route [58] cannot be rejected. However, haplogroup X was not found in any of the 21 ancient Sardinian samples reported by Olivieri et al. [15].

### Haplogroup T2b3

Sample MS10560, from BEY 197, was identified as a T2b3 with an additional mutation at position 151. T2b is a haplogroup that we did not identify in any of the modern Lebanese we sequenced though T2 is found at low frequencies there today and was identified in one of our modern Lebanese samples. Haplogroup T2 is currently found at around 10% frequency in northern and central Europe, though some of the highest rates are in Sardinia, southern Portugal and pockets in northern Spain. T2b is the most common T2 subgroup found in Europe today and it has been identified in ancient samples from the LBK in central Europe. Haplogroup T2c is reported in an early Neolithic sample (5295–5066 calBCE) from the Els Trocs site in the Pyrenees [48]. There has been some debate regarding the origins of the T2 lineages, which were originally thought to have had a Near East origin and spread into Europe with Neolithic expansions. To date, in the ancient Near East, T2b has only been identified in two Neolithic samples from north-western Anatolia, dated to around 6500BCE [48]. It has not been found in 45 samples tested from Israel, Jordan and Iran, dating from the Natufian through the Early Bronze Age [40] or in 15 Pre-Pottery Neolithic (PPN) samples from Syria [58], or 9 PPN samples from central Anatolia [51]. It is possible that the origins of T2 and in particular T2b may indeed be in southern Central Europe in the LGM, as suggested by Pala et al. [46].

Finally, the presence of a T2b3 haplotype in an ancient sample from Lebanon is unusual, as it does not appear to be an indigenous lineage. The tomb in which the burial was found was not marked and did not have any of the artefacts found in site 198, suggesting different burial customs. This is further evidence suggesting that this individual belonged to a different socioeconomic class or ethnic group, perhaps a slave brought to Beirut from one of the Phoenician settlements. Of particular interest is the reporting of a T2b3 with the 151 mutation (plus one more mutation at position 9926) in an ancient sample (KY399146) reported by Olivieri et al. [15] dating to 3086 ± 85 calBP from Cagliari, Sardinia. Cagliari was the site of another significant Phoenician colony, Karaly. Our ancient Beirut sample is most closely related to modern samples from Tunisia (Fig 3D), again, possibly indicating movement of women through the Phoenician networks from European colonies and trading ports to Phoenician cities in the homeland and Carthage.

### Integration of indigenous Sardinians in the Phoenician/Punic society of Monte Sirai

Olivieri et al. [15] suggest that their analyses of Sardinian-Specific Haplogroups and ancient mitogenomes dating to between 6000 and 3000 years ago may indicate that the earliest inhabitants of the island arrived prior to the Neolithic expansion into the region, which is consistent with archaeological and Y-chromosome data suggesting earlier settlement [59–62]. In particular, they identify haplogroups K1a2d and U5b1i1 as likely Mesolithic arrivals in Sardinia, but also raise the possibility that several H1 and H3 subgroups found in modern Sardinians are pre-Neolithic. While we found several H lineages in our ancient Phoenicians, including a shared H3 haplotype with an ancient pre-Phoenician Sardinian sample, we found no evidence of K1 or U5 lineages in the Monte Sirai samples. Recent analyses of 3514 whole-genome sequences of modern Sardinians [63] suggest pre-Neolithic occupation of the island and that some of the populations, particularly those in the mountainous central eastern (Gennargentu) region of the island, carry higher levels of this hunter-gatherer DNA compared to populations from more accessible regions. Our result, finding no K1 or U5 mitochondrial lineages in Monte Sirai, may be indicative of early social and/or geographic isolation of those hunter-gatherer populations.

The proposed Phoenician lineages, W5, N1b1a5 and X2b are not found in Sardinians today, suggesting rare introductions, of the nature of single individuals rather than as representative of major colonizing groups. We suggest that N1b1a5 could be an introduction from Carthage or some other North African location within the Phoenician network. It is therefore interesting that Chiang et al. [63] do find evidence in the whole-genome data of a small degree of what they suggest may be either sub-Saharan ancestry or North African ancestry prior to the Arab expansions, which they indicate could indeed be related to Phoenician contact.

## Conclusions

Our analyses of ancient pre-Phoenician and Phoenician mitogenomes from Lebanon and Sardinia provide important clues on cultural expansion, assimilation and population mobility in the Mediterranean between the 5^th^ and 3^rd^ centuries BCE. First, we see a certain degree of continuity of population ancestry between Phoenician and pre-Phoenician populations in Sardinia, which is consistent with archaeological evidence of integration between the cultures [64, 65]. However, our data from Monte Sirai, combined with our previously published result identifying a European mitochondrial haplogroup, U5b2c1, in a young man buried in a Phoenician crypt in Carthage, North Africa [17], provide evidence of several instances of unexpected, non-indigenous mitochondrial haplotypes in Phoenician burials both in and outside the homeland of Lebanon. These include the T2b3 haplotype in the BEY 197 site, Beirut, Lebanon, and the Near Eastern N1b1a5 and a northern European W5 found in Monte Sirai, Sardinia. Sardinia has been the subject of numerous genetic studies due to its important geographic isolation. The Sardinian population is often described as the best representation of early farmer ancestry [40, 41], and indeed, we do see the likely early farmer mtDNA lineages combined with those of the Southwestern/Central Mediterranean Mesolithic (particularly haplogroups H and possibly H1, H3 and H5), in the Phoenician samples at Monte Sirai. Brandt et al. [45] highlight the need for more aDNA complete mitogenomes to interpret the variation within Haplogroup H in the past and to assign specific subtypes to particular regions and time periods. The seven ancient Phoenician H samples identified here can help contribute to that goal. However, we also see haplogroups that indicate other influences in the population history of Sardinia, with the appearance of N1b1a5 and W5, which have not yet been recorded in modern Sardinian populations. These two haplogroups and possibly X2b, we suggest, are examples of Phoenician introductions to Sardinia, not only from the Phoenician homeland or via Punic settlements in North Africa, but may represent the mobility of women through the wider Phoenician trade networks.

While previous Y chromosome analyses of modern populations around the Mediterranean demonstrated the impact of Phoenician males on the genetic makeup of many communities [5], we now see that it is likely that the Phoenician trade networks or settlements strategies also included the translocation of women throughout the region as well as the assimilation of indigenous women in Phoenician settlements. Analyses of genetic variation in modern Lebanese populations [6] suggest closer affiliations with Europe based on mitochondrial lineages. Our result, identifying the European lineage T2b3 in the BEY197 site, suggests that some of those European mtDNA lineages could be associated with the integration of European women in Phoenician communities in Lebanon.

Finally, numerous studies published in the last five years highlight the importance of aDNA studies in understanding population change through time, though most of these have focused on the Neolithic transition. While these studies tell a story of significant population replacement in Europe with the arrival of farmers, analyses of later historic burials associated with Roman Britain [66], for example, and now our data from Phoenician sites, demonstrate that both migration and cultural assimilation were common, resulting in surprisingly cosmopolitan communities in the past.

## Supporting information

S1 Table Modern Lebanese haplogroup assignments and Genbank accession numbers. (XLSX)

S2 Table Haplogroup assignments, coverage information and variable sites identified for modern Lebanese samples. (XLSX)

S3 Table Haplogroup assignments, coverage information, ContamMix results and variable sites identified for all ancient samples sequenced. (XLSX)

S1 Fig Aerial view of the site of Monte Sirai. (TIF)

S2 Fig Median average read depth and coverage across genomes of 14 ancient Phoenician samples. (PDF)

S3 Fig DNA fragment length distribution for each of 14 ancient Phoenician samples. (PDF)

S4 Fig DNA damage patterns for Phoenician samples. Base frequency of 5’ and 3’ of strand breaks (top) and C to T nucleotide misincorporations for the first and last 25 bases of endogenous mtDNA fragments for merged reads (bottom), red = C to T and blue = G to A misincorporation.
(PDF)

S5 Fig Maximum parsimony tree with 14 ancient Phoenician samples placed within the 438 published ancient mitogenomes shown in Figure S5 of Olivieri et al. (2017). (XLSX)
