# Supplementary figures and images for: Ancient mitogenomes of Phoenicians from Sardinia and Lebanon: A story of settlement, integration, and female mobility
Source: PLoS One. 2018 Jan 10;13(1):e0190169. doi: 10.1371/journal.pone.0190169 (PMC5761892; doi:10.1371/journal.pone.0190169)

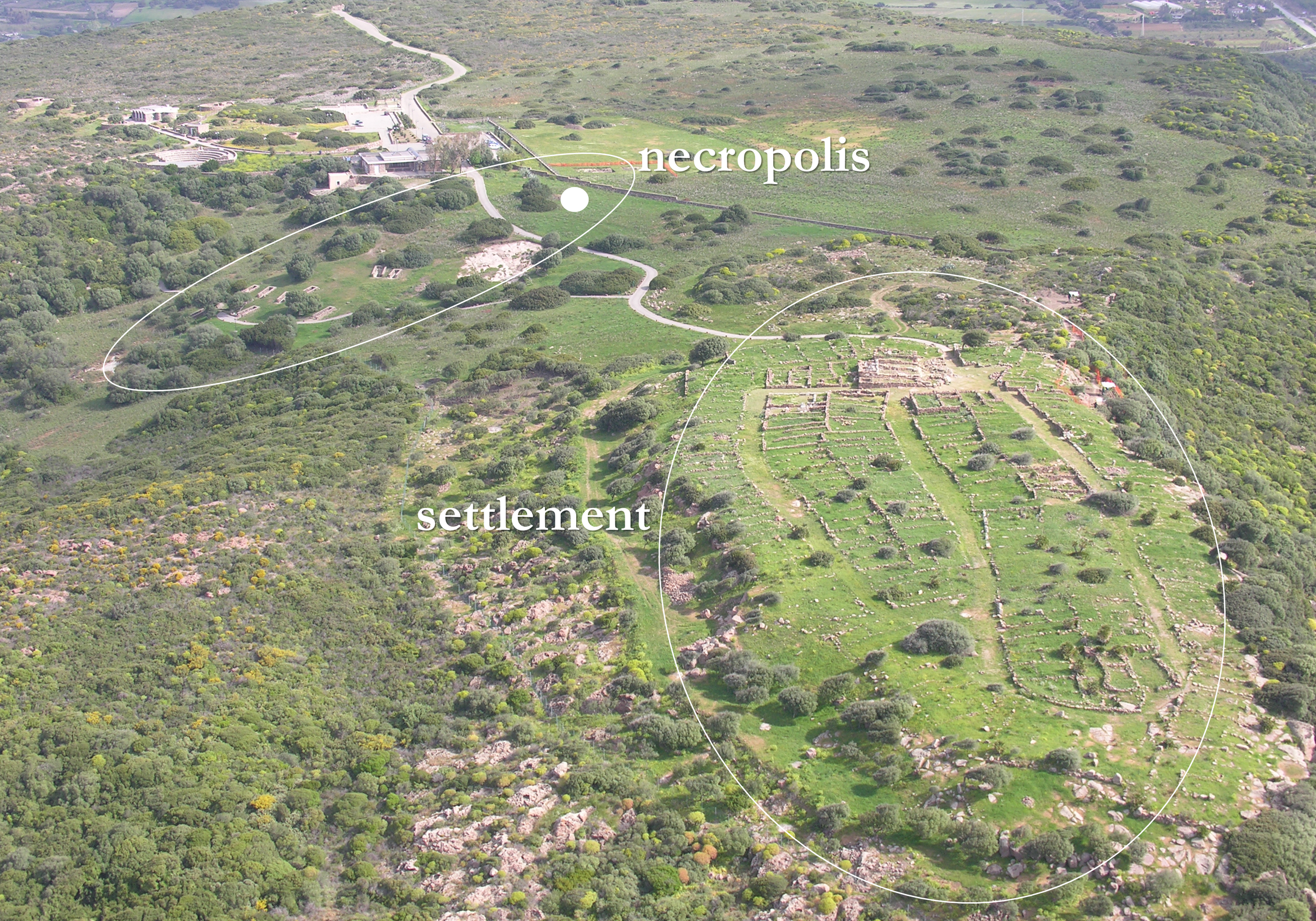

Supplement: S1 Fig — (TIF) [file pone.0190169.s004.tif]

MS10560

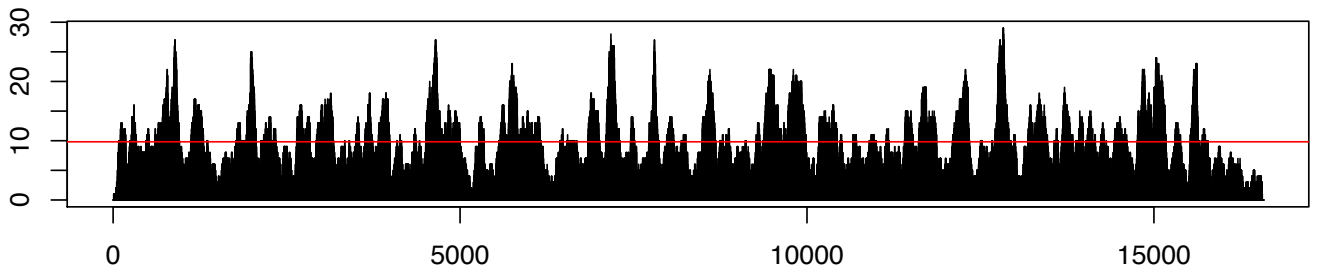

MS10562

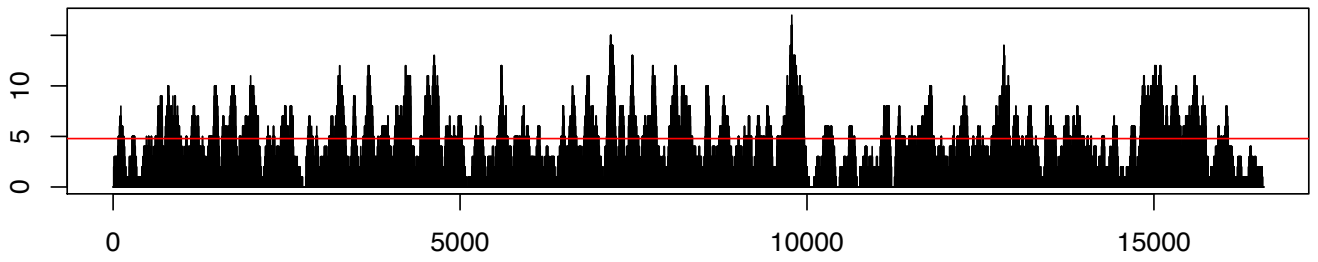

MS10565

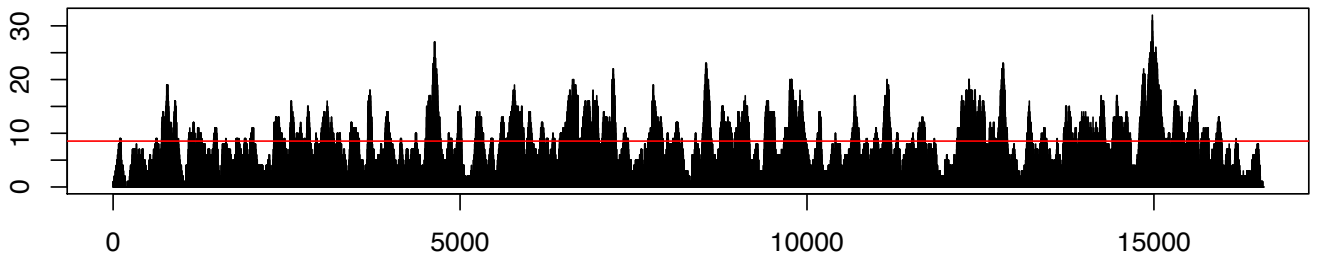

MS10575

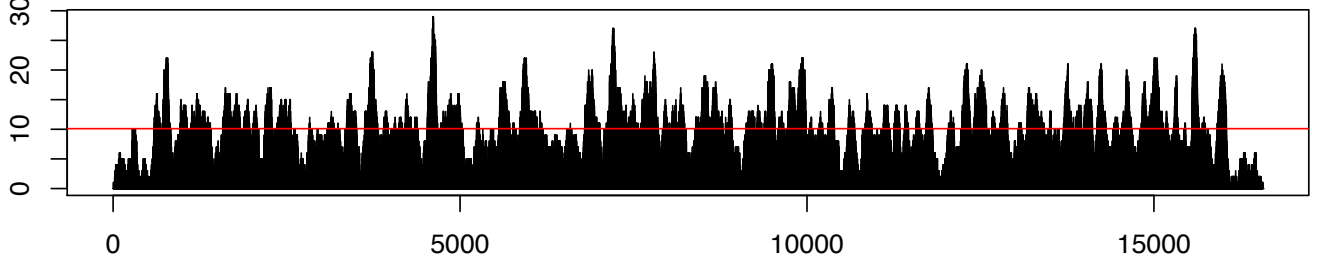

MS10577

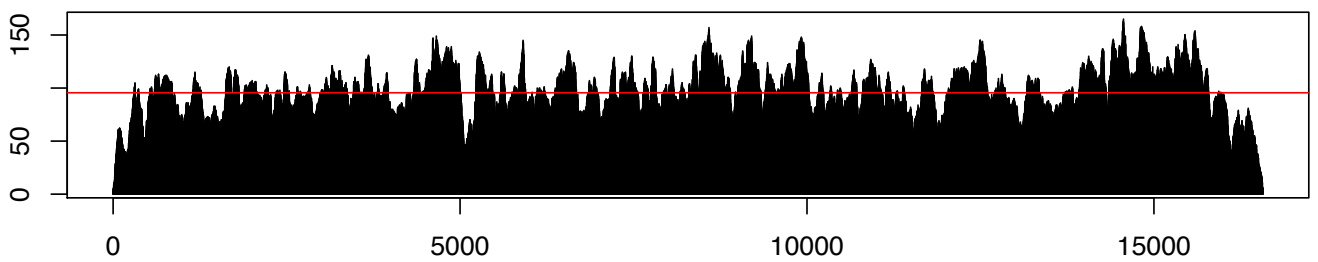

MS10578

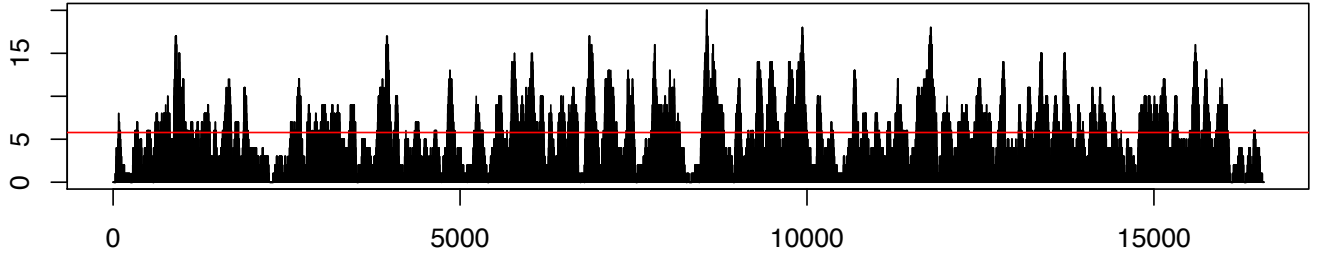

MS10579

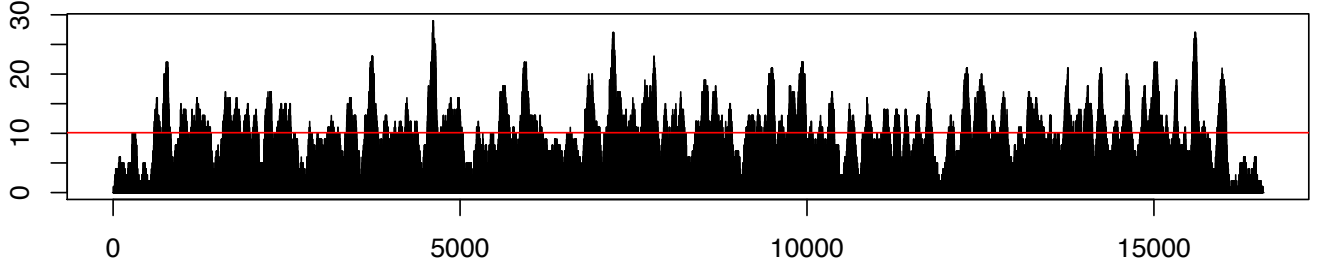

MS10580

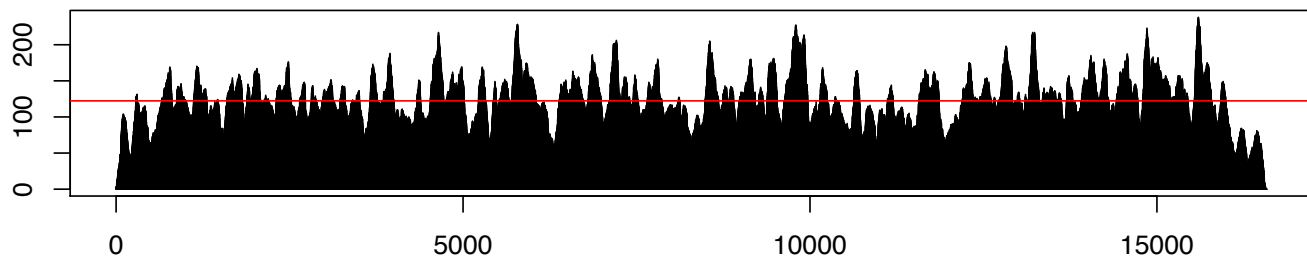

MS10581

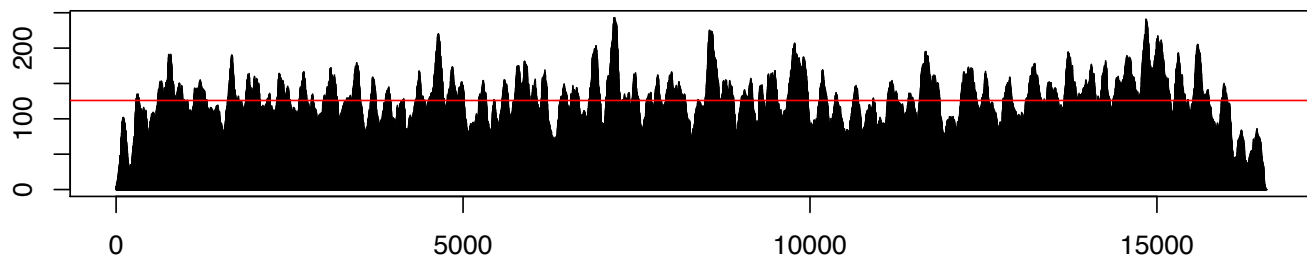

MS10582

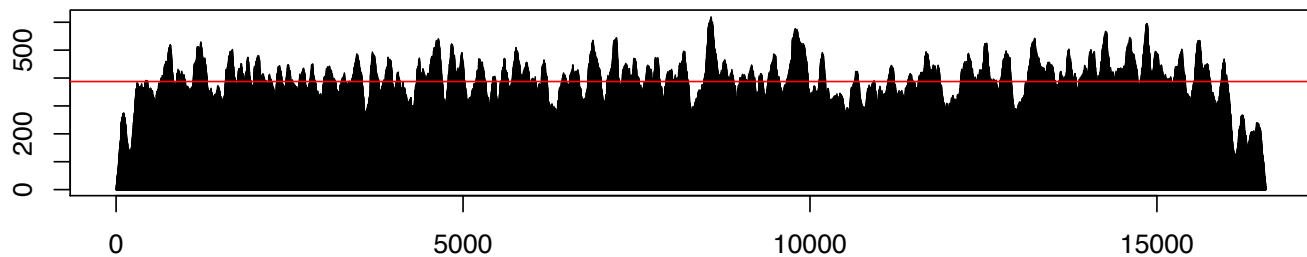

MS10584

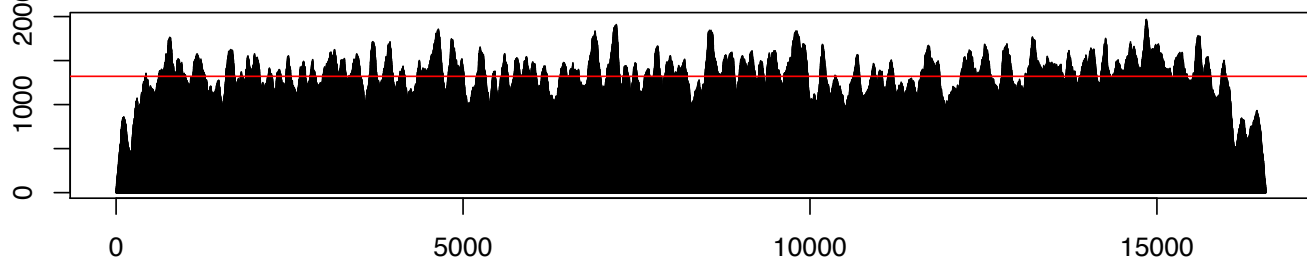

MS10585

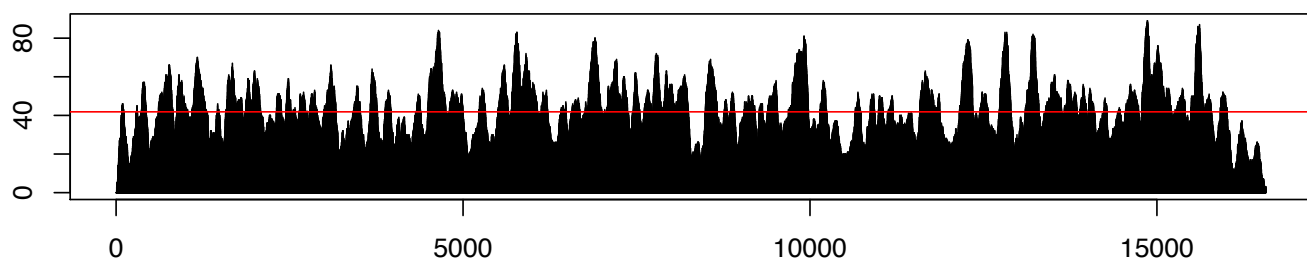

MS10587

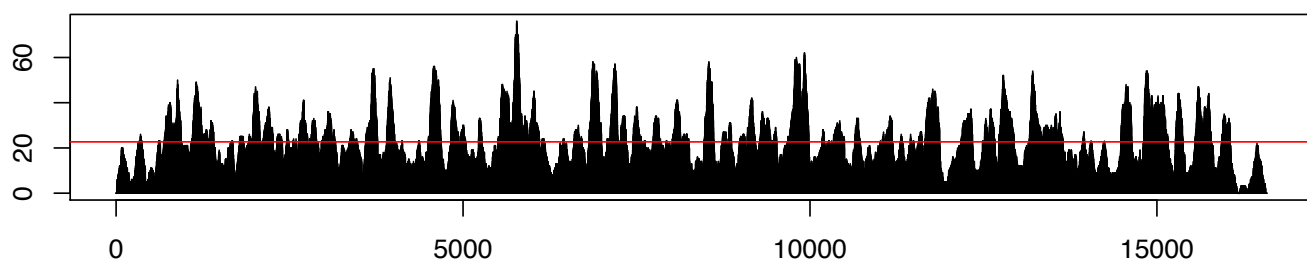

MS10588

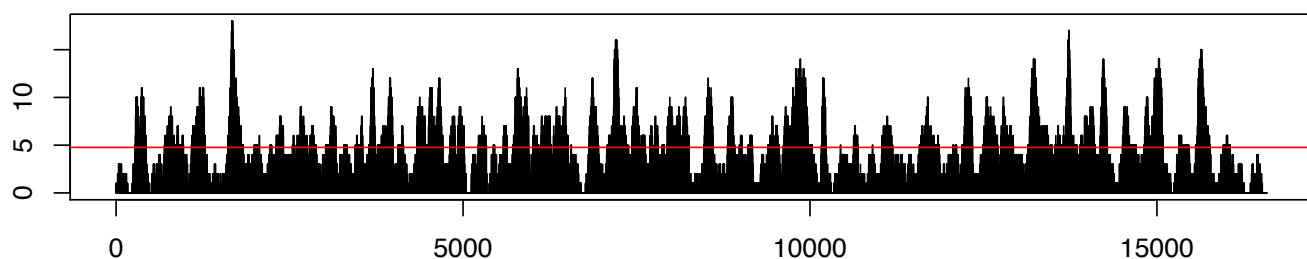

Supplement: S2 Fig — (PDF) [file pone.0190169.s005.pdf]

MS10560

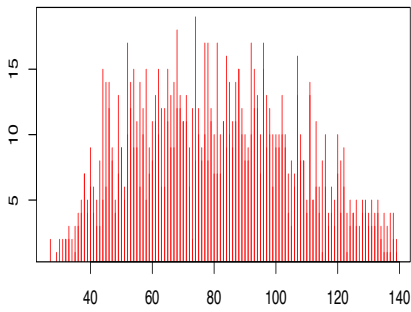

MS10562

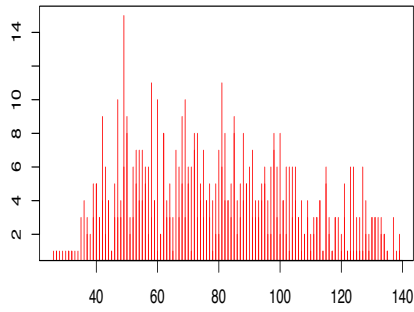

MS10565

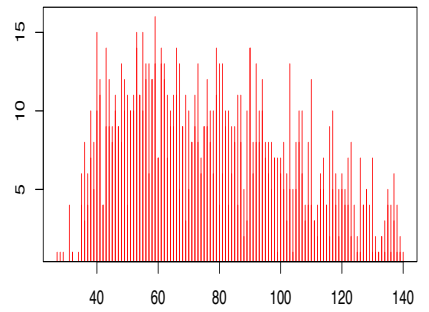

MS10575

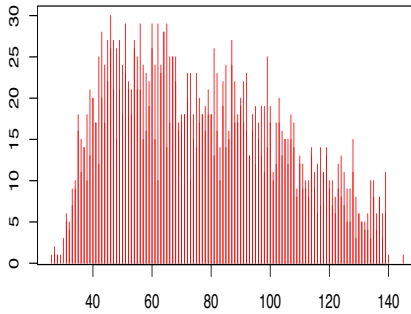

MS10577

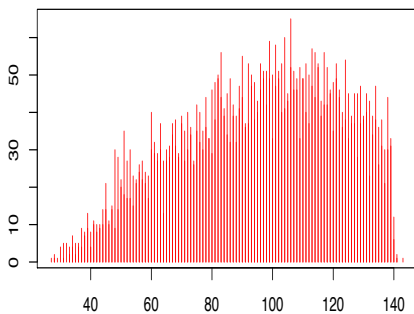

MS10578

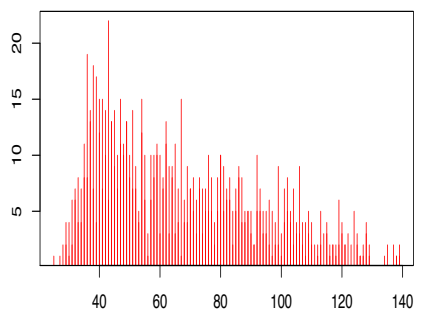

MS10579

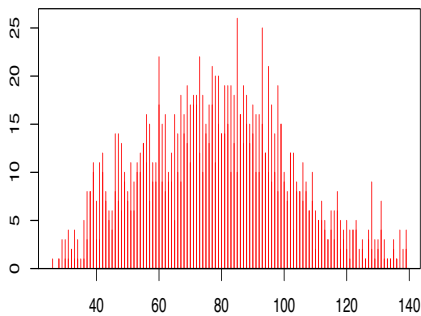

MS10580

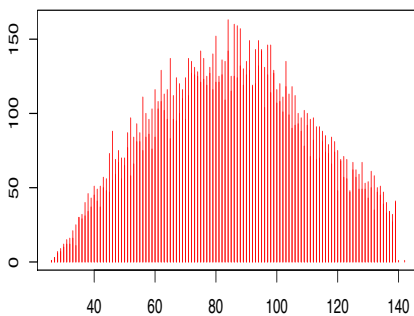

MS10581

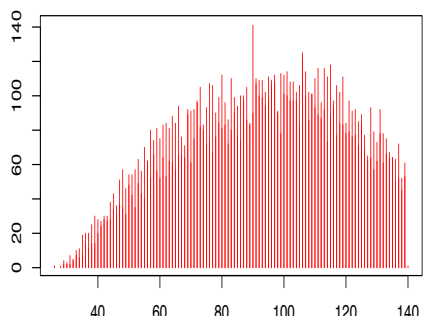

MS10582

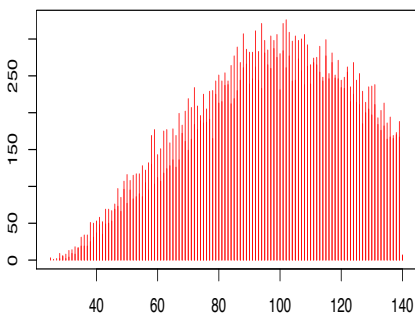

MS10584

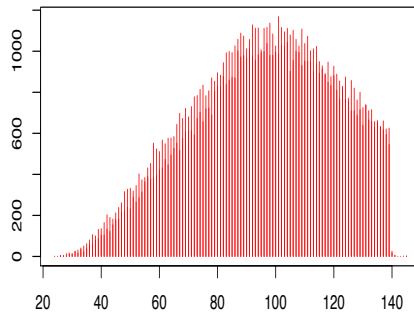

MS10585

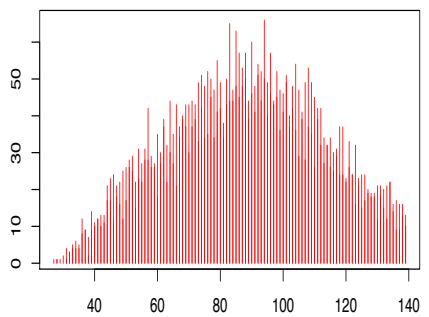

MS10587

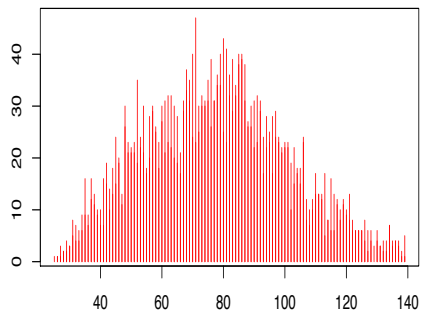

MS10588

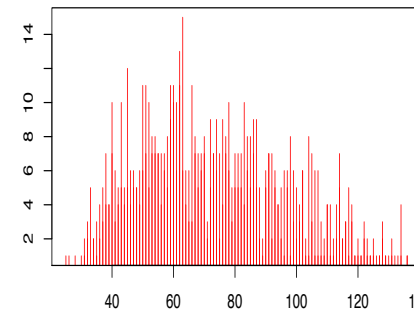

Supplement: S3 Fig — (PDF) [file pone.0190169.s006.pdf]

MS10560

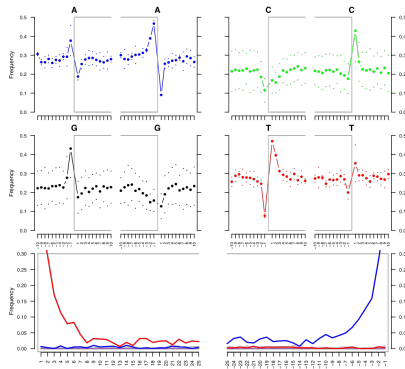

MS10562

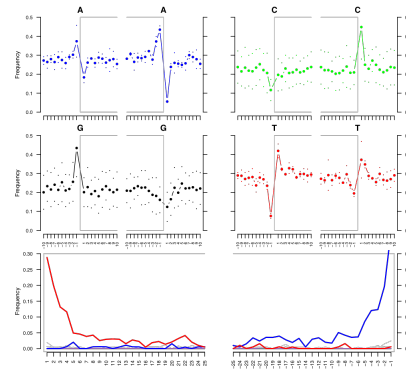

MS10565

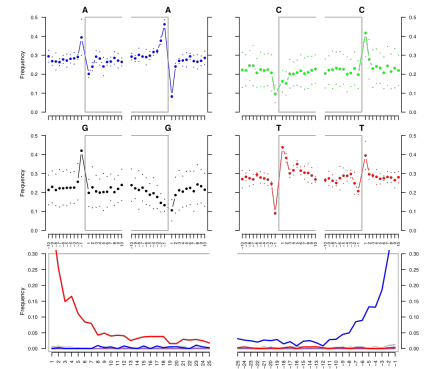

MS10575

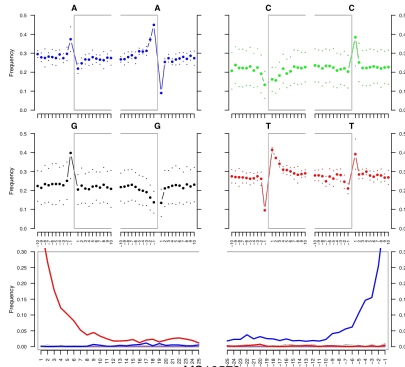

MS10577

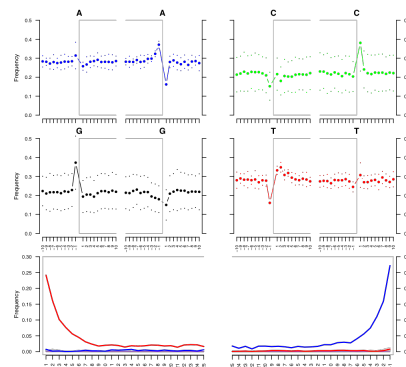

MS10578

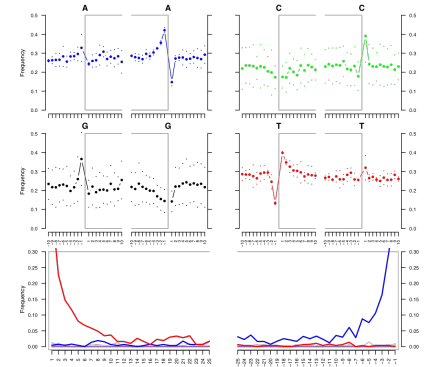

MS10579

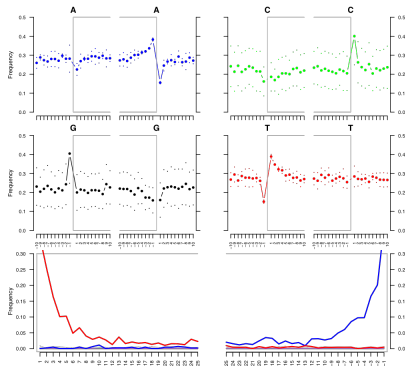

MS10580

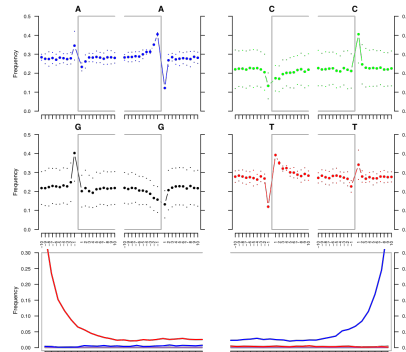

MS10581

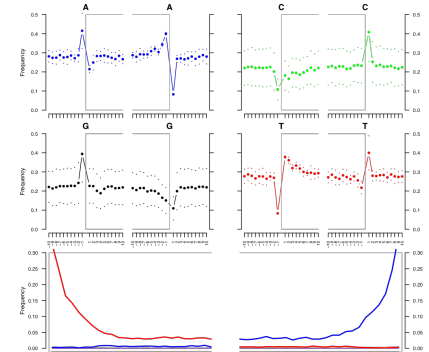

MS10582

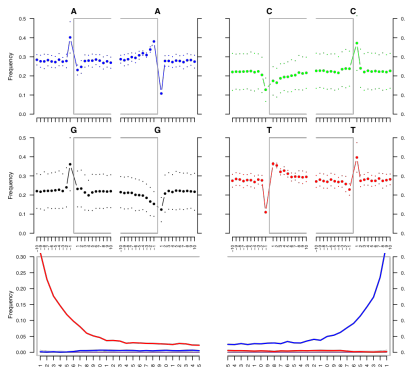

MS10584

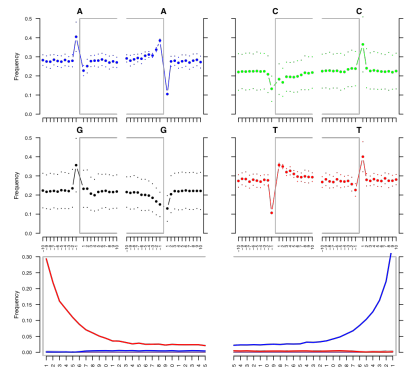

MS10585

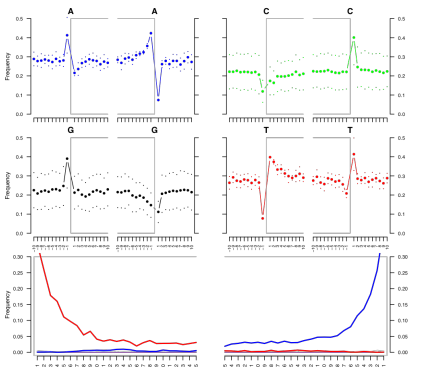

MS10587

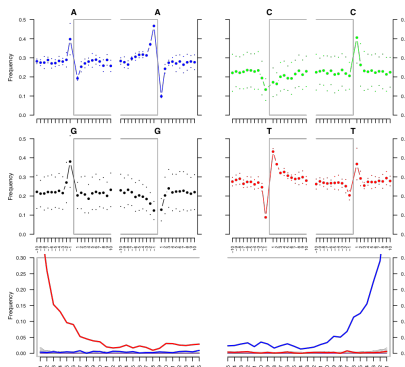

MS10588

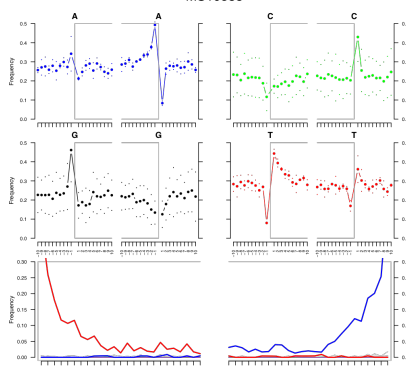

Supplement: S4 Fig — Base frequency of 5’ and 3’ of strand breaks (top) and C to T nucleotide misincorporations for the first and last 25 bases of endogenous mtDNA fragments for merged reads (bottom), red = C to T and blue = G to A misincorporation. (PDF) [file pone.0190169.s007.pdf]
